# How Augmenting Reality Changes the Reality of Simulation: Ethnographic Analysis

**DOI:** 10.2196/45538

**Published:** 2023-06-30

**Authors:** Daniel Loeb, Jamie Shoemaker, Allison Parsons, Daniel Schumacher, Matthew Zackoff

**Affiliations:** 1 Division of Critical Care Department of Pediatrics Cincinnati Children's Hospital Medical Center Cincinnati, OH United States; 2 Center for Simulation and Research Cincinnati Children’s Hospital Medical Center Cincinnati, OH United States; 3 Rescue Agency San Diego, CA United States; 4 Division of Emergency Medicine Cincinnati Children's Hospital Medical Center Cincinnati, OH United States; 5 University of Cincinnati College of Medicine Cincinnati, OH United States

**Keywords:** simulation, augmented reality, computerized mannequin, video review

## Abstract

**Background:**

Simulation-based medical education (SBME) provides key medical training for providers to safely and ethically practice high-risk events. Augmented reality (AR)–enhanced simulation projects digital images of realistic examination findings into a participant’s field of view, which allows nuanced physical examination findings such as respiratory distress and skin perfusion to be prominently displayed. It is unknown how AR compares to traditional mannequin (TM)–based simulation with regard to influencing participant attention and behavior.

**Objective:**

The purpose of this study is to use video-based focused ethnography—a problem-focused, context-specific descriptive form of research whereby the research group collectively analyzes and interprets a subject of interest—to compare and categorize provider attention and behavior during TM and AR and provide suggestions for educators looking to delineate these 2 modalities.

**Methods:**

Twenty recorded interprofessional simulations (10 TM, 10 AR) featuring a decompensating child were evaluated through video-based focused ethnography. A generative question was posed: “How do the attention and behavior of participants vary based on the simulation modality?” Iterative data collection, analysis, and pattern explanation were performed by a review team spanning critical care, simulation, and qualitative expertise.

**Results:**

The attention and behavior of providers during TM and AR simulation clustered into three core themes: (1) focus and attention, (2) suspension of disbelief, and (3) communication. Participants focused on the mannequin during AR, especially when presented with changing physical examination findings, whereas in TM, participants focused disproportionately on the cardiorespiratory monitor. When participants could not trust what they were seeing or feeling in either modality, the illusion of realism was lost. In AR, this manifested as being unable to physically touch a digital mannequin, and in TM, participants were often unsure if they could trust their physical examination findings. Finally, communication differed, with calmer and clearer communication during TM, while AR communication was more chaotic.

**Conclusions:**

The primary differences clustered around focus and attention, suspension of disbelief, and communication. Our findings provide an alternative methodology to categorize simulation, shifting focus from simulation modality and fidelity to participant behavior and experience. This alternative categorization suggests that TM simulation may be superior for practical skill acquisition and the introduction of communication strategies for novice learners. Meanwhile, AR simulation offers the opportunity for advanced training in clinical assessment. Further, AR could be a more appropriate platform for assessing communication and leadership by more experienced clinicians due to the generated environment being more representative of decompensation events. Further research will explore the attention and behavior of providers in virtual reality–based simulations and real-life resuscitations. Ultimately, these profiles will inform the development of an evidence-based guide for educators looking to optimize simulation-based medical education by pairing learning objectives with the ideal simulation modality.

## Introduction

For over 20 years, simulation-based medical education (SBME) has demonstrated clear benefits across a wide range of fields, including pediatrics [1], cardiology [2], and surgery [3]. Further, trainees can practice high-risk procedures and review rare pathology without subjecting patients to risk, an ethical imperative [4]. In aggregate, the benefits of SBME have reached the bedside, resulting in improved patient care [5].

The growth of SBME runs countercurrent to the declining role of bedside clinical training. Bedside teaching has decreased, by some accounts, from 78% of total teaching time in the 1970s [6] to 17% in the mid-2000s [7]. Whether this is due to more administrative duties, shorter lengths of stay [8,9], increasing patient complexity, or growing physician discomfort with bedside teaching [10], the end result is less time spent learning at the bedside from experts.

These challenges have created space for SBME to expand its role. Novel simulation modalities such as augmented reality (AR) and immersive virtual reality (VR) have brought with them the promise of introducing nuanced physical examination findings to the simulated bedside [8,11]. However, new does not necessarily mean better. Before we can intelligently invest the time, energy, and resources into these emerging technologies, we must learn how they impact the simulated environment and, subsequently, learner attention and behavior, so that these nascent technologies may be optimally applied to medical education. Does controlling what trainees see in a clinical scenario influence how they perceive it? The aim of this study was to identify and categorize provider attention and behavior during traditional computerized mannequin (TM)–based and AR-enhanced SBME to inform suggestions for educators looking to delineate these 2 modalities.

## Methods

### Study Design

We used video-based focused ethnography [12,13] to study a cohort of video-recorded TM and AR simulations. This approach allowed the primary research group to explore the data corpus with a focused research question [12]: “How do the attention and behavior of participants vary based on the simulation modality?” During this focused exploration, the team moved from (1) identifying and classifying the data to (2) description and analysis to (3) pattern explanation [13,14].

### Data Corpus

A series of interprofessional TM and AR simulations were reviewed. All sessions portrayed a decompensating 8-year-old with progressive shock that leads to cardiac arrest. The sessions took place in a fully functional simulation laboratory with cardiorespiratory monitors, respiratory escalation devices, a fully stocked crash cart, and all the other supplies typical of an intensive care unit (ICU; Multimedia Appendix 1). A SimJunior mannequin (Laerdal) was used for both modalities. The AR simulation added a realistic virtual pediatric patient overlay, corresponding to the dimensions of the mannequin that dynamically changed throughout the scenario. Via a mobile headset platform, the virtual patient overlay portrayed key clinical findings, including mental status (ranging from conversant to altered), perfusion (mottled skin that progressed to poor perfusion and cyanosis), and respiratory status (superimposed retractions, tachypnea, and eventually apnea; Multimedia Appendix 2). A detailed description of this AR simulation was previously described by Zackoff et al [8].

Video data were collected and stored using SimulationIQ software (Education Management Software), processed and compiled using Adobe Premiere Elements (Adobe), annotated via Vimeo (Vimeo), and coded in Excel (Microsoft Corp). Each simulation had 3 video feeds—one from the foot of the bed (typically behind the team leader), one over the patient bed (nearest to the nurse and the respiratory therapist [RT]), and one capturing the cardiorespiratory monitor. The multiple audiovisual feeds allowed for data triangulation [15], capturing the perspectives of different participants in the room.

### Participants

**Figure 1:**
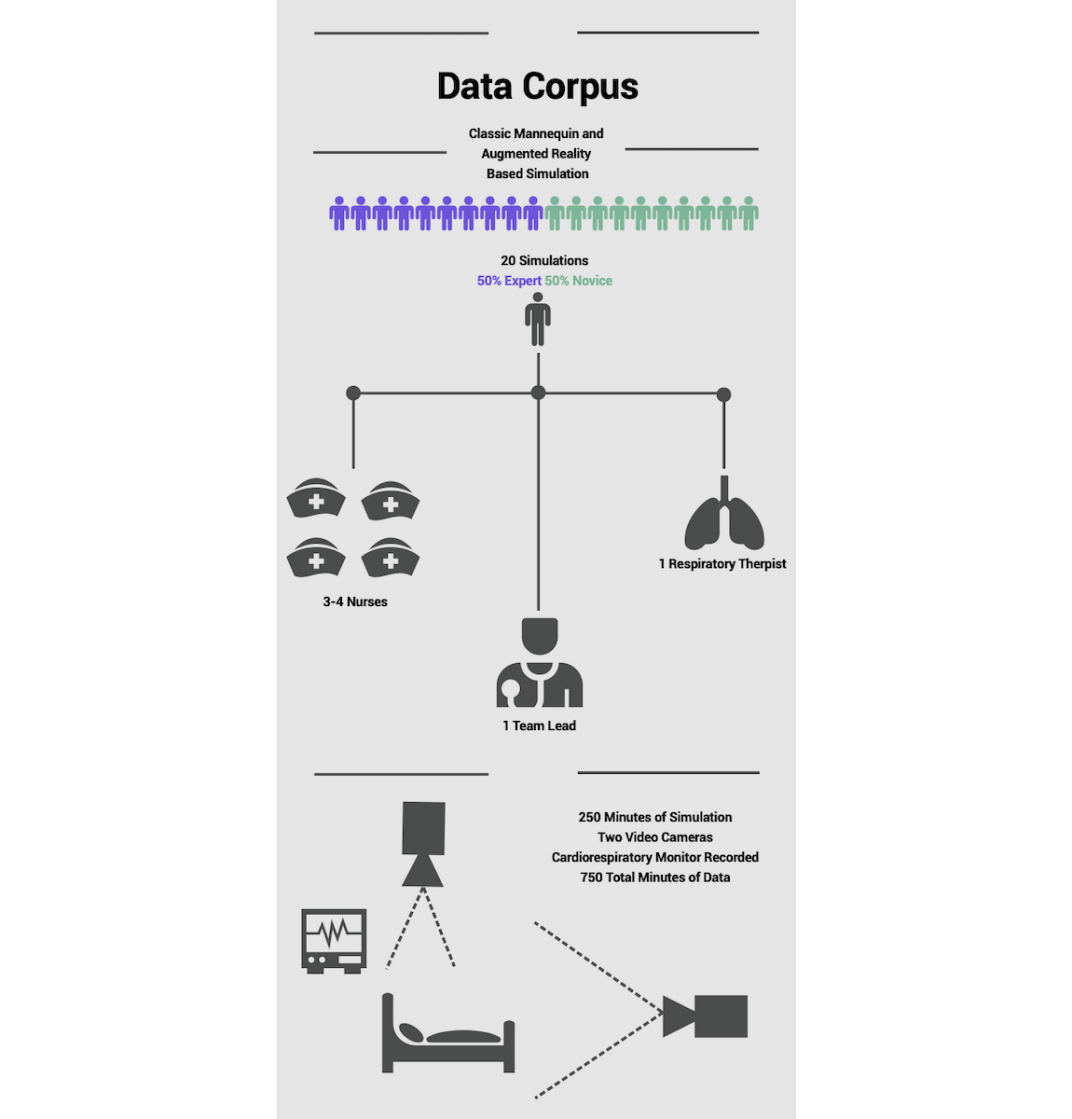
Data corpus for video review. Ten classic mannequin-based simulations and 10 augmented reality–enhanced simulations. Each simulation had 1 team lead, 3-4 nurses, and 1 respiratory therapist. A combined 750 minutes of audiovisual data were recorded by 3 cameras.

The primary research team reviewed 20 interprofessional simulations. Each simulation group was composed of a team lead physician, 3-4 nurses, and an RT. The team lead was a clinician who would traditionally lead a pediatric resuscitation team consisting of a pediatric critical care nurse practitioner, a pediatric critical care fellow physician, or a pediatric critical care attending physician. The nurses and RTs were staff from the pediatric or cardiac ICUs and served on the institution’s code response team. Each group ran a TM and an AR simulation. A total of 250 minutes of simulation sessions were analyzed using 750 minutes of recorded video (Figure 1).

### Data Analysis Team

Considering reflexivity and the desire for analytic triangulation [16] among the primary research team, we composed a heterogeneous group of experts in critical care (DL), simulation (JS), and qualitative methods (AP). DL is a practicing pediatric critical care physician as well as a simulation educator. JS is a full-time simulation educator and former pediatric emergency department nurse. AP is a qualitative researcher who specializes in human interactions and communication. A fourth reviewer, MZ, oversaw the data analysis. He is a pediatric critical care physician and education scientist who has designed, implemented, and evaluated SBME using novel modalities such as VR and AR. He met with the team at scheduled intervals and when consensus confirmation was needed by the primary research team.

### Analysis-Focused Research Question

The research team proposed the following generative question: “How do the attention and behavior of participants vary based on the simulation modality?”

### Data Analysis

**Figure 2:**
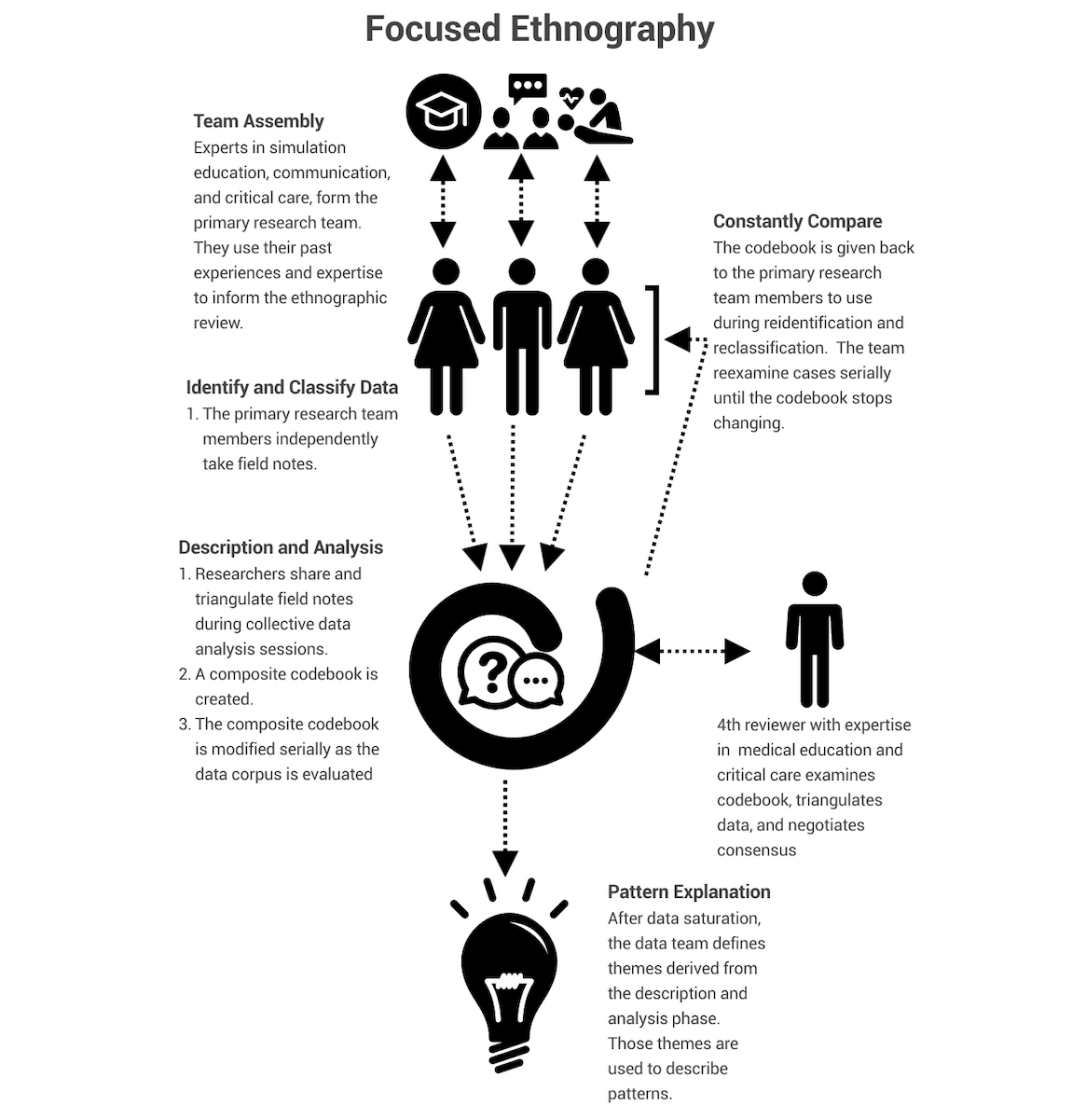
Description of the stepwise focused video-based ethnographic method.

To address this question, the TM and AR simulations were reviewed and iteratively coded through three phases (Figure 2): (1) identification and classification, (2) description and analysis, and (3) pattern explanation.

In the initial identification and classification phase, a small number of the simulations were sampled in parallel by our primary research team. The researchers were tasked with familiarizing themselves with the scenarios, the environment, and the technology and to begin taking field notes (ie, observations timestamped to points in the video by the research team) [17]. After the initial data sampling period, the primary research team took field notes independently. Examples of field notes include transcriptions of participant statements, observations related to the positioning and focus of the team, and other points of interest recognized by the researchers. These independently generated field notes were treated as data and shared during collective data analysis sessions (Multimedia Appendix 3). During these sessions, the primary research team met to reconcile differences in independent coding via triangulation between the team members [15] and to negotiate consensus for the generation of a composite codebook [18].

After the collective data analysis sessions, the primary research team independently applied the composite codebook to the simulation sessions. After reanalyzing each simulation session, the group reconvened and modified the codebook as needed. This process of data description and (re)analysis via constant comparative analysis [19] continued until the data reached saturation, after 20 interprofessional sessions. Subsequently, the primary research team (DL, AP, and JS) sorted the categories in the composite codebook into themes while considering the generative question, “How does the attention and behavior of participants vary based on the simulation modality?” Themes were created by reviewing the codebooks and identifying repeating patterns of attention and behavior among the participants that spanned across multiple reviewed scenarios. The major themes were aggregated and summarized to illustrate provider attention and behavior during TM and AR simulations and subsequently triangulated by MZ. Finally, these themes allowed for a comparative description and pattern explanation of the strengths and weaknesses of these simulation modalities.

### Ethical Considerations

The primary study and this secondary analysis were reviewed by the Cincinnati Children’s Hospital Institutional Review Board (study ID: 2019-0210) and received a waiver of documentation of informed consent per 45 CFR 46.116(d), which allows the institutional review board to approve a waiver of documentation of consent for research that involves no more than minimal risk to subjects, does not affect the rights and welfare of subjects, could not practicably be carried out without the waiver, and if possible, the subjects will be provided additional pertinent information after participation. This study met the criteria given its educational nature with no risk to participants.

Participation in the simulations was voluntary, with no compensation offered. All information regarding participant performance was stored on a password-encrypted server. All participants provided documented consent to filming, with videos stored on a password-protected server.

## Results

### Overview

**Figure 3:**
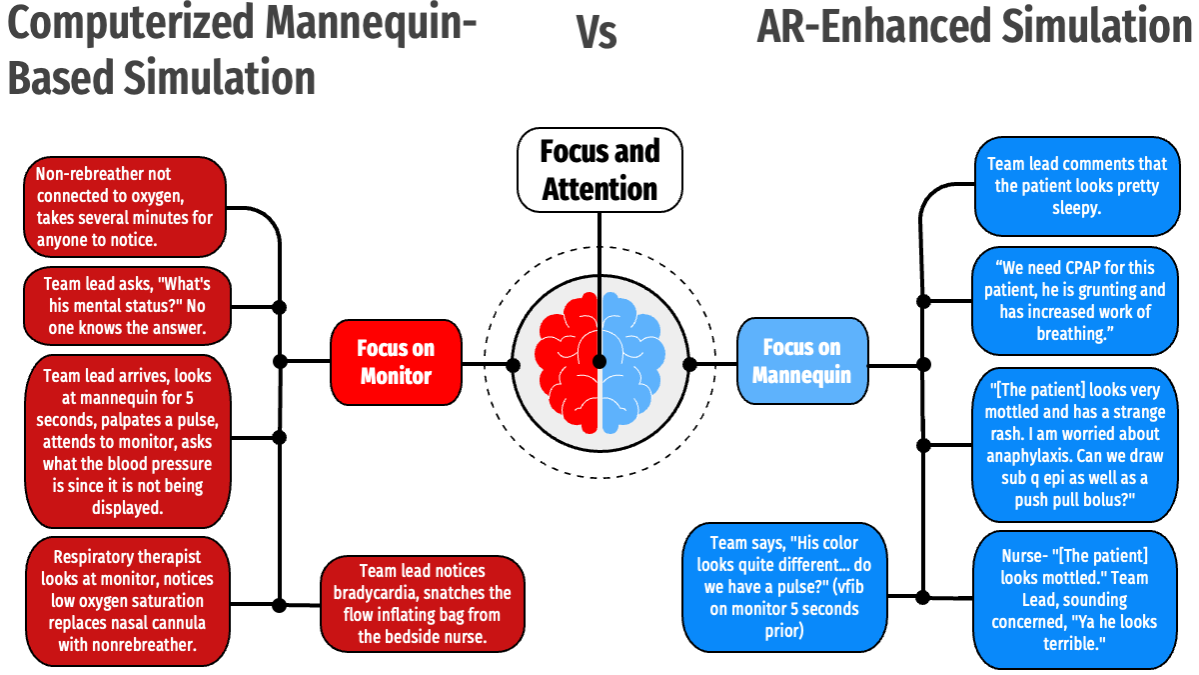
Main theme 1, “Focus and Attention,” with associated subcategories and illustrative quotes and examples. AR: augmented reality; CPAP: continuous positive airway pressure.

**Figure 4:**
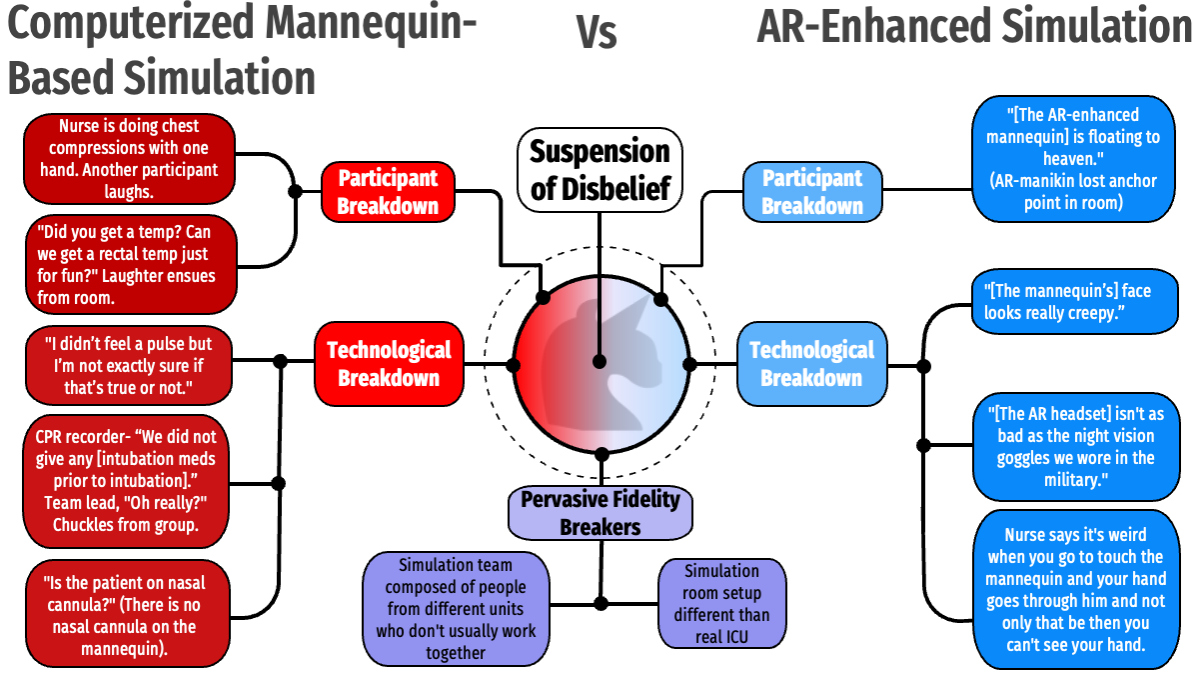
Main theme 2, “Suspension of Disbelief,” with associated subcategories and illustrative quotes and examples. AR: augmented reality; CPR: cardiopulmonary resuscitation; ICU: intensive care unit.

**Figure 5:**
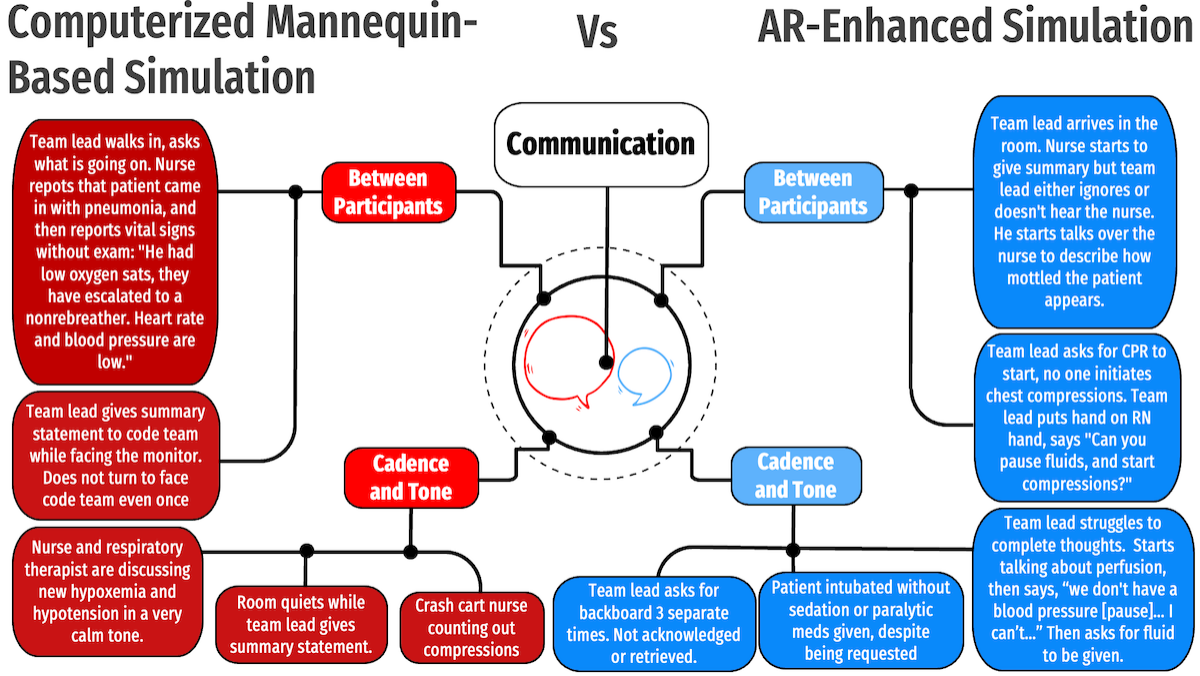
Main theme 3, “Communication,” with associated subcategories and illustrative quotes and examples. AR: augmented reality; CPR: cardiopulmonary resuscitation.

Pattern explanation generated 3 core themes and associated subthemes (Figures 3-5). Theme 1, “Focus and Attention,” included two subthemes: (1) focus on the monitor and (2) focus on the mannequin. Theme 2, “Suspension of Disbelief,” included three subthemes: (1) breakdown from technology, (2) breakdown from participants, and (3) pervasive fidelity breakers. Theme 3, “Communication,” included two subthemes: (1) communication character between participants and (2) room cadence and tone.

### Theme 1: Focus and Attention

The simulated scenario offered participants multiple sensory inputs in parallel, which required the participants to triage and process those inputs. Participant focus varied between the 2 modalities with regards to being primarily on the cardiorespiratory monitor in TM simulations versus the mannequin in AR simulations.

In all observed simulations, participants focused on the most dynamic or reliable source of information. In the TM simulation, this manifested as participants neglecting the mannequin and prioritizing treatment based on data from the cardiorespiratory monitor. For example, the RTs were unlikely to fully auscultate the patient. Instead, they noted hypoxemia on the monitor and placed the patient on a nasal cannula without touching or listening to the patient with any sincerity. Reliance on the cardiorespiratory monitor was shared by the team lead who would listen to the story upon arrival but attend primarily to the monitor. Anything more than a cursory physical examination (eg, palpating a femoral pulse) was rare in the TM simulations.

In the AR simulations, the virtual patient overlaying the mannequin dynamically changed as the case evolved. This dynamic appearance led to a shift in participant focus toward the mannequin. This shift in focus often influenced management, with the team lead noting patient work of breathing and color as justification for initiation of continuous positive airway pressure as opposed to simply choosing a nasal cannula to address hypoxemia conveyed by the vital sign monitor. In one scenario, the skin findings conveyed by the virtual patient overlay, in combination with the visible dyspnea, prompted team concern for and treatment of anaphylaxis rather than septic shock.

### Theme 2: Suspension of Disbelief

Both modalities allowed for episodes where the illusion of realism was lost. We found that these “fidelity-breakers” could be divided into (1) technological breakdown, (2) participant breakdown, and (3) pervasive fidelity-breakers, which were those issues that existed across both TM and AR simulation.

Technological breakdowns were situations in which the participants could not trust what they were seeing or feeling. During TM simulations, participants would often attempt an examination maneuver (eg, feel for a pulse) but question the accuracy of their findings. The participants would look to the facilitator for affirmation or request that the “correct” examination be provided (eg, the examiner says to the facilitator, “I didn’t feel a pulse but I’m not exactly sure if that’s true or not.”). AR simulation ameliorated some, but not all, of the technological breakdowns that occurred in TM simulation. It was rare for participants in AR simulations to solicit information from the facilitator about mental status, perfusion, or respiratory status. Instead, participants made statements such as, “Wow, this patient looks terrible,” followed by recommendations for the next steps (eg, push-pull a fluid bolus). The enhanced audiovisual and psychological-cognitive fidelity [20] of the AR simulation, such as a visible breathing pattern and perfusion changes, allowed participants to overcome residual distrust in their examination of the mannequin.

However, technological breakdowns also occurred in the AR simulations, which interfered with participants’ ability to interface with the world. Specifically, several participants were disoriented and therefore hesitant to move while wearing the headset. Though 1 provider commented that the headset was “better than the night vision goggles we used in the military,” several others described a variety of motion sickness side effects (dizziness, blurry vision, nausea). In a small minority, motion sickness became intolerable. The AR technology sometimes malfunctioned, projecting the virtual patient a few inches above the physical mannequin, which made physical interactions with the mannequin challenging. For example, the RTs often struggled to find the mannequin’s mouth and would just pantomime, assisting ventilation.

Participant behavioral breakdowns were defined as participant statements or actions that significantly impacted the team’s ability to suspend disbelief. These behaviors were most apparent during physical interactions with the mannequin during the TM simulation. These participant breakdown behaviors were less common during the AR simulations, with strong engagement in the patient’s clinical assessment as the patient declined.

Last, pervasive fidelity breakers transcended both simulation modalities. The simulation room itself was not identical to the institution’s ICU rooms, and the team makeup included mixed staff from the pediatric and cardiac ICUs. Equipment retrieval time and the subsequent speed of clinical interventions were affected.

Following all types of fidelity breakers, participants would often speak hypothetically without acting. During a representative example, a participant turned to the facilitator and said, “I would usually put oxygen on the patient at this time,” but then did not apply oxygen. These types of fidelity-breaking events are not unique to this simulation and are prevalent in SBME [21].

### Theme 3: Communication

Interprofessional communication was a key driver of decision-making during the scenarios. Communication was subcategorized into (1) communication character between participants and (2) room cadence and tone.

Early communication occurred between participants while they were identifying the principal problem. A team member would assess the patient and then corroborate that assessment with the group (eg, “[The mannequin] sounds diminished” or “I am having trouble feeling a pulse too”). As additional participants were called into the room, they were oriented to the scenario by summary statements delivered by already-present participants. This new, larger group then generated consensus opinions regarding examination findings and subsequent management. Participants during the TM scenarios maintained eye contact, used physical touch, and engaged in 2-way communication. In AR scenarios, providers often stabilized the AR headsets with their hands and moved around the room slowly. These behaviors limited the amount of eye contact and physical communication possible.

In both modalities, providers relied on each other for examination consensus. However, the content of the consensus was different. In the TM simulation, participants were more likely to discuss vital signs such as worsening hypoxemia, bradycardia, and hypotension. In the AR simulations, participants discussed physical examination findings, such as perfusion and neurologic status.

The tone and cadence of the room intensified as the patient worsened in both modalities. The slow need for escalation of care at the start of the scenarios afforded the participants time to recruit additional staff. As the number of participants increased, so did the acuity of the patients. In TM simulation, this escalating acuity manifested as a more concerning cardiorespiratory monitor with a discordantly static patient appearance. In the AR simulation, the mannequin also appeared sicker, which informed management. This focus on the poor appearance of the patient led to an intensification of the tone and cadence of the room. In this heightened environment, participants missed details, interrupted each other, and failed to engage in closed-loop communication frequently—unlike during the TM simulations, which allowed for calm closed-loop communication throughout.

## Discussion

### Principal Findings

We used video-based focused ethnography to expose the variations in clinician attention and behavior during TM and AR simulations. Though prior research has examined quantitative metrics in simulation (eg, time to cardiopulmonary resuscitation [CPR] and quality of chest compressions), we are unaware of other attempts to scrutinize the events antecedent to those kinds of outcome metrics. These discoveries provide an alternative methodology to categorize simulation, shifting focus from modality and fidelity to participant behavior and experience.

For the TM simulation, participants focused on reliable sources of information and avoided those they could not trust. This distrust affected participants’ confidence in examination findings. Consequently, participants skipped portions of the examination altogether, such as checking perfusion or neurologic status. Participants responded to cardiorespiratory monitor changes by escalating oxygen therapy, administering intravenous fluids, and initiating CPR, all without consideration for the patient’s examination otherwise. Participants effectively engaged in these key management tasks, performing them as they would in real-life clinical care. These findings suggest that TM simulation may be the optimal tool for teaching practical skill acquisition while remaining limited for training or evaluating clinical assessment skills or behaviors. Finally, TM simulation routinely resulted in a calm room with strong 2-way communication and frequent eye contact. Therefore, this modality may be better suited for introducing the core skills and behaviors required during a code response to novice learners.

The behaviors in AR simulation, alternatively, were defined by the enhanced visual and cognitive fidelity introduced by the AR virtual patient overlay and the requisite technological costs to facilitate it. The AR-enhanced mannequin prominently displayed many physical examination findings—specifically mental status, perfusion, and work of breathing—transforming it into a reliable data stream for participants. This shifted focus to the mannequin from the cardiorespiratory monitor, facilitating the enhanced ability for training on and evaluation of clinical assessment skills. Though participants focused on and responded to dynamic physical examination findings in the AR environment, they struggled with procedural tasks (ranging from applying oxygen to high-quality CPR). Finally, the AR simulations were associated with environments that appear more aligned with real-life experiences—loud and chaotic, with missed communication occurring frequently. This more realistic cadence and sense of urgency could be valuable for training and assessing more experienced clinicians.

To understand the ramifications of our findings, it is important to consider the limitations of our approach. First, our data was taken from a single institution over a narrow period and consisted of 20 simulations, a relatively small sample size. However, the participants represent a large sample of the pediatric code response team at a large academic medical center, so the behaviors may be similar at other large pediatric institutions. Additionally, the data reached saturation after 20 scenarios were reviewed, suggesting that a review of additional scenarios would not have yielded new findings. Second, focused ethnography is an inductive form of research, meaning that the experiences and expertise that the researchers bring to the data analysis are intrinsic to the methodology and strengthen the analysis by adding richness to the drawn conclusions. This research team, with expertise in simulation and resuscitation, was deliberately assembled to review the cases and inject their perspectives into the data, enriching the interpretation and strengthening the analysis.

Finally, the scenarios occurred sequentially, with the TM simulation followed by the AR simulation. Though the scenarios did not progress identically, their temporal relationship precludes our team from directly quantifying differences in clinical performance metrics. Regardless, the focused research question sought to explore provider attention and behavior as a consequence of the technology used, not the specifics of participant clinical performance. Descriptions of other novel simulation modalities, comparisons between other institutions, and quantifiable clinical performance metrics all represent future key pursuits of this investigative team. The learnings from this study inform which quantifiable metrics (eg, total noise volume in the room, percentage of closed-loop communication, recognition of arrhythmia) might be modifiable via AR simulation.

### Conclusions

This study characterized participant attention and behavior in both TM and AR simulations. Through video-based focused ethnography, 3 key themes emerged: focus and attention, suspension of disbelief, and communication. Our findings provide an alternative methodology to categorize simulation, shifting focus from simulation modality and fidelity to participant behavior and experience. This alternative categorization suggests that TM simulation may be superior for practical skill acquisition and the introduction of communication strategies for novice learners, while AR simulation offers the opportunity for advanced training in clinical assessment. Further, AR simulation could be a strong communication and leadership training tool for more experienced clinicians due to the generated environment being more representative of decompensation events. The next steps include exploring participant behaviors in completely digital training experiences, such as VR. Finally, we aim to compare participant behaviors during all these simulation modalities to true patient encounters. Collectively, these endeavors will inform the development of an evidence-based guide for educators looking to optimize SBME by pairing identified learning objectives with the ideal simulation modality, ultimately leading to improved patient care.

## Appendix

Multimedia Appendix 1 Nurse Practitioner, bedside Nurse, and Respiratory Therapist discussing the status of a patient in augmented reality.

Multimedia Appendix 2 Augmented reality overlay visible to the participants.

Multimedia Appendix 3 Excerpt from the identification and classification phase, whereby independent field notes were taken by the research team and then compiled via Vimeo software. The blue dot represents the location of the code in the video and is timestamped accordingly.

## Abbreviations

AR: augmented reality
CPR: cardiopulmonary resuscitation
ICU: intensive care unit
RT: respiratory therapist
SBME: simulation-based medical education
TM: traditional mannequin
VR: virtual reality

## Data Availability

The data sets generated and analyzed during the study are not publicly available due to the video consent stipulating that the video collected cannot be used outside this research study and due to individual participant identifiers used in the field notes. However, a sample of deidentified field notes is available from the corresponding author upon reasonable request to assist with the illustration of the study methodology.

## References

[1] Lopreiato JO, Sawyer T (2015). Simulation-based medical education in pediatrics. Acad Pediatr.

[2] McKinney J, Cook DA, Wood D, Hatala R (2013). Simulation-based training for cardiac auscultation skills: systematic review and meta-analysis. J Gen Intern Med.

[3] Stefanidis D, Scerbo MW, Montero PN, Acker CE, Smith WD (2012). Simulator training to automaticity leads to improved skill transfer compared with traditional proficiency-based training: a randomized controlled trial. Ann Surg.

[4] Ziv A, Wolpe PR, Small SD, Glick S (2003). Simulation-based medical education: an ethical imperative. Acad Med.

[5] Zendejas B, Brydges R, Wang AT, Cook DA (2013). Patient outcomes in simulation-based medical education: a systematic review. J Gen Intern Med.

[6] Ahmed MEBK (2002). What is happening to bedside clinical teaching?. Med Educ.

[7] Crumlish CM, Yialamas MA, McMahon GT (2009). Quantification of bedside teaching by an academic hospitalist group. J Hosp Med.

[8] Zackoff MW, Cruse B, Sahay RD, Fei L, Saupe J, Schwartz J, Klein M, Geis GL, Tegtmeyer K (2021). Development and implementation of augmented reality enhanced high-fidelity simulation for recognition of patient decompensation. Simul Healthc.

[9] Nair BR, Coughlan JL, Hensley MJ (1997). Student and patient perspectives on bedside teaching. Med Educ.

[10] Janicik RW, Fletcher KE (2003). Teaching at the bedside: a new model. Med Teach.

[11] Zackoff MW, Real FJ, Sahay RD, Fei L, Guiot A, Lehmann C, Tegtmeyer K, Klein M (2020). Impact of an immersive virtual reality curriculum on medical students' clinical assessment of infants with respiratory distress. Pediatr Crit Care Med.

[12] Higginbottom GM, Pillay JJ, Boadu NY (2013). Guidance on performing focused ethnographies with an emphasis on healthcare research. Qual Rep.

[13] Andreassen P, Christensen MK, Møller JE (2020). Focused ethnography as an approach in medical education research. Med Educ.

[14] Knoblauch H, Schnettler B (2012). Videography: analysing video data as a 'focused' ethnographic and hermeneutical exercise. Qual Res.

[15] Denzin NK (1970). The Research Act in Sociology: A Theoretical Introduction to Sociological Methods.

[16] Flick U (2004). Triangulation in qualitative research. Companion Qual Res.

[17] Phillippi J, Lauderdale J (2018). A guide to field notes for qualitative research: context and conversation. Qual Health Res.

[18] Knoblauch H (2005). Focused ethnography. Forum Qual Soc Res.

[19] Charmaz K (2006). Constructing grounded theory: a practical guide through qualitative analysis. Nurse Res.

[20] Hancock PA, Vincenzi DA, Wise JA, Mouloua M (2008). Human Factors in Simulation and Training.

[21] Liu D, Macchiarella ND, Vincenzi DA, Vincenzi DA, Wise JA, Mouloua M, Hancock PA (2008). Simulation fidelity. Human Factors in Simulation and Training.

