# Supplementary figures and images for: How Augmenting Reality Changes the Reality of Simulation: Ethnographic Analysis
Source: JMIR Med Educ. 2023 Jun 30;9:e45538. doi: 10.2196/45538 (PMC10365567; doi:10.2196/45538)

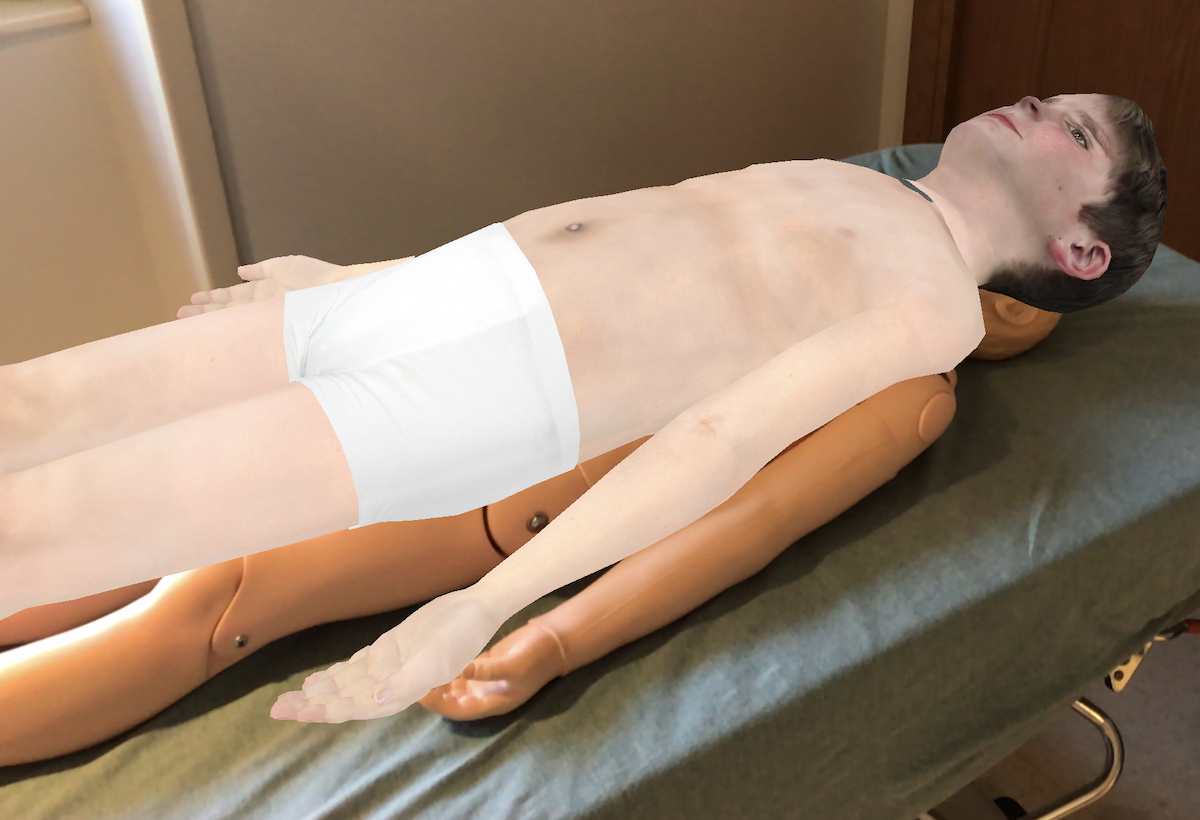

Supplement: Multimedia Appendix 2 [file mededu_v9i1e45538_app2.png]

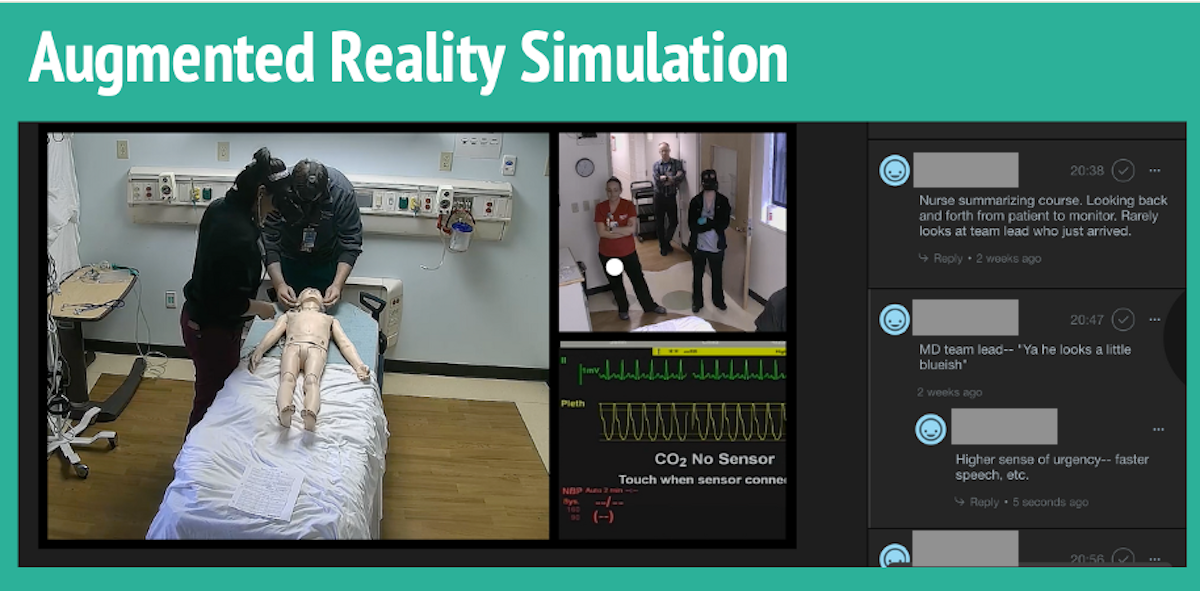

Supplement: Multimedia Appendix 3 [file mededu_v9i1e45538_app3.png]
